# The Inflammatory Role of Platelets via Their TLRs and Siglec Receptors

**DOI:** 10.3389/fimmu.2015.00083

**Published:** 2015-03-02

**Authors:** Fabrice Cognasse, Kim Anh Nguyen, Pauline Damien, Archibald McNicol, Bruno Pozzetto, Hind Hamzeh-Cognasse, Olivier Garraud

**Affiliations:** ^1^Etablissement Français du Sang Auvergne-Loire, Saint-Etienne, France; ^2^GIMAP-EA3064, Université de Lyon, Saint Etienne, France; ^3^Faculty of Health Sciences, Colleges of Pharmacy and Medicine, University of Manitoba, Winnipeg, MB, Canada; ^4^Institut National de Transfusion Sanguine (INTS), Paris, France

**Keywords:** platelets, innate immunity, cytokine/chemokine, inflammation, TLR, Siglec

## Abstract

Platelets are non-nucleated cells that play central roles in the processes of hemostasis, innate immunity, and inflammation; however, several reports show that these distinct functions are more closely linked than initially thought. Platelets express numerous receptors and contain hundreds of secretory products. These receptors and secretory products are instrumental to the platelet functional responses. The capacity of platelets to secrete copious amounts of cytokines, chemokines, and related molecules appears intimately related to the role of the platelet in inflammation. Platelets exhibit non-self-infectious danger detection molecules on their surfaces, including those belonging to the “toll-like receptor” family, as well as pathogen sensors of other natures (Ig- or complement receptors, etc.). These receptors permit platelets to both bind infectious agents and deliver differential signals leading to the secretion of cytokines/chemokines, under the control of specific intracellular regulatory pathways. In contrast, dysfunctional receptors or dysregulation of the intracellular pathway may increase the susceptibility to pathological inflammation. Physiological vs. pathological inflammation is tightly controlled by the sensors of danger expressed in resting, as well as in activated, platelets. These sensors, referred to as pathogen recognition receptors, primarily sense danger signals termed pathogen associated molecular patterns. As platelets are found in inflamed tissues and are involved in auto-immune disorders, it is possible that they can also be stimulated by internal pathogens. In such cases, platelets can also sense danger signals using damage associated molecular patterns (DAMPs). Some of the most significant DAMP family members are the alarmins, to which the Siglec family of molecules belongs. This review examines the role of platelets in anti-infection immunity via their TLRs and Siglec receptors.

## Introduction

It is well accepted that blood platelets play a principal role in primary hemostasis. Since more than 50 years, they have been used therapeutically as transfused blood products (1), and platelet extracts have been used for their healing properties, especially in ophthalmology (2). Platelet concentrate transfusions were considered necessary in a number of situations (e.g., central thrombocytopenia in preference to peripheral thrombocytopenia; thrombopathy associated with bleeding; severe bleeding or risk of bleeding) (3). However, transfusion hazards and, at minimum, discomfort, are common (4), leading the medical community to question this therapy.

Traditionally, hemostasis and thrombosis have been considered difficult to understand for non-specialists. However, there is a general understanding that human platelet antigen (HPA) polymorphisms can complicate the post-transfusion situation in thrombopathies, such as von Willebrand disease (4) and pregnancies and deliveries [notably regarding fetal neonatal allo-immunization (FNAIT) (5)], along with allo-immunization to HLA class I antigens responsible for transfusion refractoriness (6).

Now, however, it is recognized that platelets have a complex role in the whole process of hemostasis and thrombosis (7). As the platelet proteome started to be investigated, it became clear that platelets contain proteins beyond those with hemostatic functions, including angiogenic factors, growth factors, pro-inflammatory factors, anti-inflammatory factors, and biological response modifiers (BRMs) (8).

## An Overview of the Main Platelet Receptors

Platelets play a vital role in hemostasis, especially primary hemostasis, and subsequently in vascular repair (9). Platelet membrane integrins interact with the subendothelial matrix of a damaged vascular wall, leading to their activation and subsequent creation of a platelet thrombus together by fibrinogen (Fg) to close the vascular lesion and terminate blood loss (10).

Activated platelets liberate arachidonic acid from membrane phospholipids, which in turn is converted to thromboxane A_2_ (TxA_2_). As a consequence, TxA_2_ is secreted and acts on TP receptors on the cell surface of other platelets, leading to further platelet activation. The TP receptor belongs to the major agonist receptor family seven transmembrane G-protein coupled receptors (GPCRs).

Granule contents are released from activated platelets. The dense granules contain important agonists like ADP and serotonin. ADP is a significant amplifier of initial platelet aggregation (11). It interacts with specific extracellular membrane receptors to induce intracellular signaling. There are two classes of receptors for ADP, P2Y1 and P2Y12, which belong to the GPCR family (12) and P2 × 1, which belongs to the ADP/ATP determined calcium channel family of purinergic receptors (13, 14). Engagement of the TP receptor by TxA_2_ and P2Y1 by ADP lead to the hydrolysis of phosphatidylinositol-4,5 bisphosphate by phospholipase C (PLC), leading to the generation of diacylglycerol (DAG) and inositol triphosphate (IP_3_) (15). DAG and IP_3_ stimulate protein kinase C and cause release of calcium from the dense tubular system, respectively, which in turn are responsible for many of the final event associated with platelet activation, including the exocytosis of platelet granules. ADP interacts with calcium activated pathway. P2Y12 receptor has been reported to potentiate platelet secretion and be involved in “sustained irreversible platelet aggregation” (12).

Platelets express a class of cell surface protease-activated receptors (PARs), which are members of the GPCR family and activated by thrombin. Human platelets only express PAR1 and PAR4, not PAR2 and PAR3 (16). PAR1 is also the most abundant PAR, expressing about 2500 copies per platelet and is, therefore, predominant for thrombin mediated platelet activation. PAR1 is a highly glycosylated protein that consist of 425 amino acids and has a molecular weight of 70 kDa. PAR4 on the other hand, the last PAR to be discovered, consists of 397 amino acids (17). The PAR1 activating peptide SFLLRN and PAR4 activating peptide AYPGKF are synthetic peptides with the same sequence as their respectively N-terminal after cleavage. They are widely used in research because they effectively and specific activate their receptors (18).

**Figure 1:**
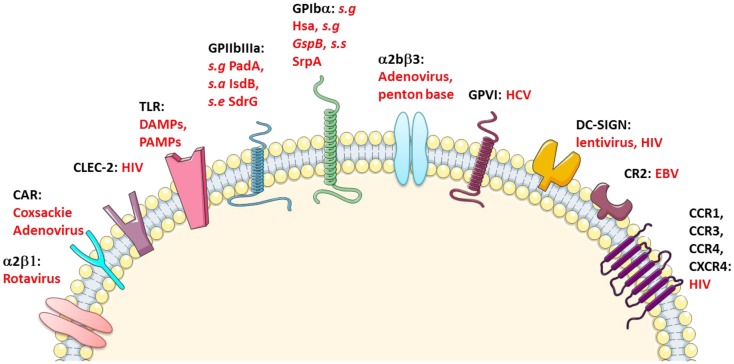
**Platelet immune receptors**. CLEC-2, C-type lectin-like type II transmembrane receptor; CR-2, complement receptor type 2; CCR-1, CCR-3, and CCR-4, C–C chemokine receptor type 1, 3, and 4, respectively; CXCR-4, C-X-C chemokine receptor type 4; DC-SIGN, dendritic cell-specific intercellular adhesion molecule-3-grabbing non-integrin; GP-VI, glycoprotein VI; CAR, coxsackie adenovirus receptor; *s.a*, *Staphylococcus aureus*; *s.g*, *Streptococcus gordonii*; *s.s*, *Streptococcus sanguinis*; *s.e*, *Staphylococcus epidermidis*. Adapted from Ref. (10, 19–23).

Interestingly, several hemostatic receptors, in addition being the subject of genetic polymorphisms leading to variant HPAs, bind to infectious pathogens (Figure 1).

Platelets also express receptors that indirectly bind infectious pathogens: Complement (C) Rs and Fc-Ig-Rcs (Table 1).

**Table 1:** **Platelet–bacteria interactions adapted from Ref. (24–26)**.

Platelet receptors	Bacteria/fungi	Bacterial proteins	Intermediary plasma molecules
GP-IIb–IIIa	*S. epidermidis*	SdrG	Fg
	*S. Aureus*	FnbpA/B	Fibronectin
	*S. Aureus*	FnbpA/B	Fg
	*S. Aureus*	ClfA	Fibronectin
	*S. Aureus*	ClfA	Fg
	*S. Aureus*	IsdB	Direct
	*S pyogenes*	M1	Fg
	*S gordonii*	PadA	Direct
	*S. lugdunensis*	Fbl	Fg
GP-Iba	*S. sanguis*	SrpA	Direct
	*S. gordonii*	GspB/Hsa	Direct
	*S. Aureus*	Protein A	vWF
	*H. pylori*	?	vWF
FCγRIIa	*S. Aureus*	FnbpA/B	IgG
	*S. Aureus*	ClfA	IgG
TLR2	*S. pneumoniae*	?	Direct
	*?*	Lipoprotein	Direct
TLR4	*E. coli*	LPS	Direct
gC1q-R	*S. sanguinis*	?	C1
TLR	Fungi	?	Complement
Protease-activated receptors	Fungi	?	Complement

*ClfA, clumping factor A; Fnbp, fibronectin-binding protein; Fg, Fibrinogen; GspB, glycosylated streptococcal protein B; Hsa, hemagglutinin salivary antigen; IsdB, iron-regulated surface determinant B; LPS, lipopolysaccharide; PadA, platelet adhesion binding protein A; SdrG, serine–aspartate repeat G; SrpA, serine-rich protein A; TLR, toll-like receptor; VWF, von Willebrand factor; ?, undefined*.

The apparently complex relationships between platelets and infectious pathogens, as well as the involvement of platelets during sepsis, which have been the subject of recent reviews (10, 27–33), led a number of groups to examine the possibility that platelets have dedicated roles in innate immunity and foremost in pathogen sensing. Those groups almost simultaneously described, both in mice and humans, the presence and then the functionality of toll-like receptors (TLRs) on (TLR2/TLR1/TLR6 and TLR4 ± TLR9) and in platelets (TLR9) (19, 23, 24, 34–36). It is only recently that TLR3 (37) and TLR7 (38) have also been identified. This discovery allowed a paradigm change in the understanding of platelet physiopathology, putting platelets in a continuum of immunity, at the crossroad of innate and adaptive immunity (10, 35, 39–41).

By contrast, Montrucchio et al. (42) demonstrated that platelets neither bind FITC-LPS nor express the LPS-receptors CD14 and toll-like receptor 4 (TLR4); in contrast, LPS primed monocytes – and to a lesser extent polymorphonuclear neutrophils – proved to adhere to platelets. Next, both platelet–leukocyte interactions and platelet aggregation (in whole blood) were inhibited by blockade of CD14 and TLR4.

It must also be noticed that the Sabroe team observed no modulation in platelet response after engagement of TLR1 and TLR4 (43), no platelet aggregation, no increase in CD62p on the platelets surface, and no increase in intra-platelet Ca2^+^ levels after stimulation by “natural” ligands of TLR2 (e.g., Pam3CSK4) or TLR4 (e.g., LPS); this allowed them to conclude that agonists of platelet TLRs have no direct effect on platelet activation. This was rather puzzling because agonists of TLR2 and TLR4 are acknowledged to play a significant role in diseases such as atherosclerosis; a mechanism other than the direct activation of platelets is believed to be involved.

Similar data have been observed by Jayachandran et al. (44) who attempted to determine whether LPS affects platelet phenotype after TLR4 engagement. After injecting knockout (KO) mice for the TLR4 coding gene with a non-lethal dose of LPS (0.2 mg/kg IV), they demonstrated that the platelets in TLR4 KO mice are less abundant than in normal mice, contain less RNA, and express less CD62p upon thrombin exposure. However, platelets from TLR4 KO mice do neither aggregate nor secrete adenosine triphosphate upon thrombin stimulation. One week after injection (which is the time required for a full turnover of the platelet pool), the number of circulating platelets, the CD62p expression, and the platelet aggregation had increased; consequently, the platelet phenotype observed 1 week after exposure to LPS appears independent on TLR4. It is concluded that the 1-week lasting effect of LPS is related to the megakaryocytes themselves and the platelets they produce, rather than to the already circulating platelets (44).

Platelets, already identified as secretors of pro-inflammatory cytokines, chemokines, and BRMs, such as soluble-CD40-Ligand (sCD40L)/sCD154, were revisited for their role in inflammation (45–48).

Initially, platelet linked-inflammation was recognized in pathologies, such as transfusion associated hazards or adverse events, however, further work also identified platelets in peripheral pathologies, such as in cardio-vascular disease and atheroma-plaque formation, inflammatory bowel disease, and arthritis (20, 27, 41, 49–58). This identified the role of platelets in pathological inflammation; however, a role for platelets in physiological inflammation, as this concept potentially established in the immunology landscape (59, 60), remained ill-defined. A series of dedicated studies aimed at stimulating platelets with a variety of defined activators with small differences demonstrated that platelets, despite being non-nucleated, are capable of mobilizing a signalosome differentially, and to secrete discrete panels of BRMs upon activator-dependent stimulation. Platelets appear to have the ability to decipher between vessel endothelial cell injuries eliciting hemostatic responses and between distinct infectious pathogen moieties; however, both types of receptors can be engaged in parallel as – for example – responses to TLR2 engagement on platelets involves PAR1 (61, 62).

In summary, the newly proposed paradigm on platelet pathophysiology is that platelets are equipped with multiple receptors aimed at sensing their environment; basically, the principal danger that can be sensed by circulating platelets is created by endothelial damage. Under normal situations, platelets can repair this damage by eliciting a local micro-inflammatory response aimed at recruiting repairing and healing molecules. In addition, platelets can also sense, under certain circumstances, other potential dangers, such as infectious pathogens harboring Pathogen Associated Molecular Patterns (PAMPs). Therefore, platelets can be regarded as sentinels of danger, especially at the vascular level. Platelets are thus involved along the spectrum of inflammation, from physiology to pathology.

Platelets are engaged in complex relationships with leukocytes [all types (63–66)], notably with polymorphonuclear cells (PMNCs – formally known as neutrophils), both in physiology and pathology (67–73). It has recently been shown that platelets express a (or the) triggering receptor expressed on myeloid cells 1 (TREM1)-receptor which, when engaged by a protein expressed on neutrophils, TRIM1, increases the activation of these cells and modulates the inflammatory response by augmenting both IL-8 secretion and the production of reactive oxygen species (ROS) (74).

Further studies identified that platelets were capable of leaving the circulation (they were initially thought to be strictly contained in the blood flux), especially by being “cargoed” by leukocytes (66, 75, 76), and to participate in peripheral pathology [by themselves and by microparticles (PMPs) shed from their membrane (77–79). The capacity of platelets to sense damaged tissues was then addressed and proved to depend on platelet expression of damage associated molecular patterns (DAMP)-sensors] (80, 81). Moreover, Varki et al. (82) proposed “self-associated molecular patterns” (SAMPs), which would be recognized by intrinsic inhibitory receptors, to maintain the baseline non-activated state of innate immune cells and dampen their reactivity following an immune response. To detect such SAMPs, there must be cognate Self-PRRs (SPPRs). The first-studied example was factor H and the second are the Siglec (sialic acid recognizing Ig-like lectins), which have N-terminal V-set Ig-like domains that recognize sialic acids and often have tyrosine-based inhibitory signaling motifs within their cytosolic tails (83–87). Siglec molecules (Siglec-7, 9, and 11) are now acknowledged as controlling platelet apoptosis (88).

## The Platelet Toll-Like Receptors

### Background on TLRs

Toll-like receptors are expressed either on the external surfaces or in the cytoplasms of a wide variety of cells involved in immune processes, and are, to a large extent, involved in natural (or innate) immunity. In particular, TLRs are membrane receptors for pathogens known to play a major role in phagocytosis and inflammation.

It should also be noted that TLRs have endogenous ligands with the capacity to cause, or accelerate, inflammation; these include heat shock proteins (HSP) 60 and 70, Fg, and a diverse products of apoptotic cells (89).

Toll-like receptor expression was initially discovered on immune cells (Table 2), such as macrophages, dendritics cells (DCs), and, later, in B-lymphocytes, as well as some categories of T lymphocytes. These receptors are key-players in the defense of the body, as they form the interface between recognition of the danger signal (pathogenic or endogenous) and initiation of various types of immune response (inflammation, release of molecules involved in inducing adaptive response). The example that best emphasizes their importance is the activation of TLRs present on the surface of DCs. The signaling pathways concerned lead to the release of pro-inflammatory cytokines as well as the “up-regulation” of molecules that potentiate the function of Ag presentation (class II CMH, IL-12, CD80) and consequently the activation of T lymphocytes (90).

**Table 2:** **Summary of known mammalian TLRs and Siglecs**.

	Name	Expression	Ligands – Sialic acid linkage specificity		Name	Expression	Ligands
**Siglec-family proteins in humans**	Siglec-1 (CD169)	Mac	α2,3 > α2,6	**TLR-family proteins in humans**	TLR1	B, Mo, Mac, DCs, Plt	Multiple triacyl lipopeptides
	Siglec-2 (CD22)	B	α2,6		TLR2	Mo, Mac, N, MyDCs, Mc, Plt	Multiple glycolipids
							Multiple lipopeptides
							Multiple lipoproteins
							Lipoteichoic acid
							HSP70
							Zymosan (beta-glucan)
	Siglec-3 (CD33)	Mo, MyP	α2,6 > α2,3		TLR3	DC, B, Plt	Double-stranded RNA
							Poly I:C
	Siglec-4 (MAG)	OligoD, Schw	α2,3 > α2,6		TLR4	Mo, Mac, N, MyDCs, Mc, B, IE, Plt	Lipopolysaccharide heat shock proteins
							Fg
							Heparan sulfate fragments
							Hyaluronic acid fragments
							Nickel
							Various opioid drugs
	Siglec-5 (CD170)	N, Mo, B	α2,3		TLR5	Mo, Mac, DC, IE	Bacterial flagellin
							Profilin
	Siglec-6 (CD327)	Troph, B	α2,6		TLR6	Mo, Mac, B, Mc, Plt	Diacyl lipopeptides
	Siglec-7 (CD328)	NK, Mo, Plt	α2,8 > α2,6 > α2,3		TLR7	Mo, Mac, pDC, B, Plt	Imidazoquinoline loxoribin (a guanosine analog)
							Bropirimine
							Single-stranded RNA
	Siglec-8	Eo, Ba	α2,3 > α2,6		TLR8	Mo, Mac, DC, Mc	Small synthetic compounds; single-stranded RNA
	Siglec-9 (CD329)	Mo, N, DC, NK, Plt	α2,3 = α2,6 (prefers sulfated residues)		TLR9	Mo, Mac, pDC, B, Plt	Unmethylated CpG oligodeoxynucleotide DNA
	Siglec-10	B, Mo, Eo	α2,3 = α2,6		TLR10	Unknown	Unknown
	Siglec-11	Mac, Plt	α2,8		TLR11	Mo, Mac, LC, KC, UBE	Profilin
	Siglec-14	ND	α2,6		TLR12	NE, pDC, DC, Mac	Profilin
	Siglec-15	ND	ND		TLR13	Mo, Mac, DC	Bacterial ribosomal RNA sequence “CGGAAAGACC”

*Toll-like receptors and Siglecs bind different ligands and are expressed by different types of leukocytes or other cell types. Human B, B cells; Ba, basophils; cDCs, conventional dendritic cells; Eo, eosinophils; GRB2, growth-factor-receptor-bound protein 2; ITIM, immunoreceptor tyrosine-based inhibitory motif; Mac, macrophages; Mo, monocytes; MyP, myeloid progenitors; N, neutrophils; ND, not determined; NK, natural killer cells; OligoD, oligodendrocytes; pDCs, plasmacytoid dendritic cells; Schw, Schwann cells; Troph, trophoblasts; Plt, platelets, My DC, Myeloid dendritic cells; Mc, Mast cells; IE, Intestinal epithelium; liver cells, LC; kidney cells, KC; Urinary Bladder Epithelium, UBE; Neurons, NE. Adapted from Ref. (10, 35, 84, 86–88, 91–97)*.

Toll-like receptors are also expressed by cells considered non-immune but which occupy the interface between the outside environment and the immune system, in particular fibroblasts and epithelial cells (98).

Toll-like receptors are type 1 transmembrane proteins possessing: (i) leucine-rich ectodomains folded into β-sheets, enabling the interaction with PAMPs; (ii) a transmembrane domain; and (iii) a cytoplasmic “Toll-interleukin-1 receptor” (TIR) domain that is fundamental to signal transduction. To date, 10 TLRs have been identified in humans and 13 in mice; TLR12 and TLR13 have not been identified in the former but the latter (89). Of note, TLR11 has the unique feature of possessing a sequence recognized as a stop codon by human transcription machinery; *Toxoplasma* profilin, a ligand of murine TLR11, is recognized by humans and a truncated, but functional, form of TLR11 is, therefore, presumed to exist in the human (99).

**Figure 2:**
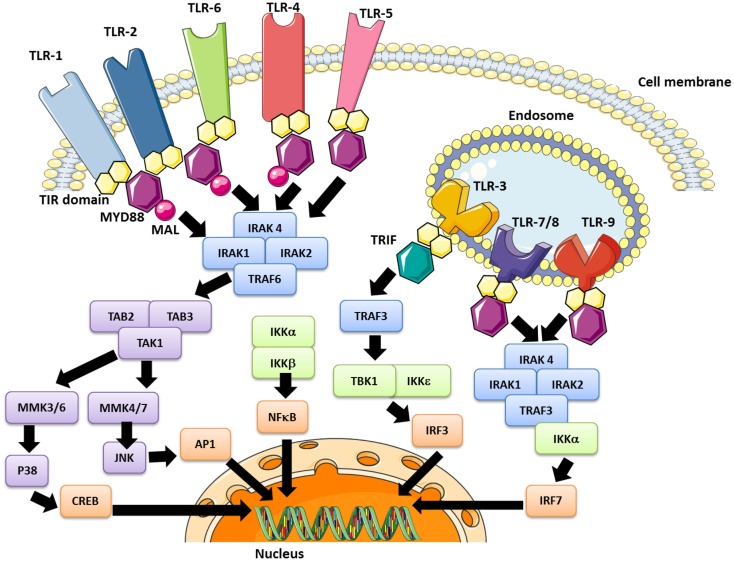
**The TLR signaling pathway and modulation effector molecules**. Depending upon the TLR involved, the nuclear translocation of transcription factors occurs, including the “nuclear factor kappa B” (NFκB) in early and late stages (all TLRs), AP-1 (all except TLR 3), the “interferon regulation factor” (IRF)-3 (TLR3 and TLR4) and IRF-7 (TLR7/8/9). These pathways lead to inflammatory cytokine synthesis, or at least secretion in the case of platelets, as well as the production of interferon type 1 (IFN1).

The PAMPs recognized by TLRs are lipids, lipoproteins, proteins, or nucleic acids derived from bacteria, viruses, fungi, or parasites. Moreover, PAMPs can be recognized by TLRs in various cellular compartments, including the plasma membrane, endosomes, lysosomes, and endolysosomes (89). After engagement, each TLR triggers its own distinctive biological response, which is specific for the PAMP recognized. These differences were identified by the discovery of various adaptive molecules that bind to the TIR domain; these include the “Myeloid differentiation primary response gene (88)” (MyD88), TIRAP, TIR-domain-containing adapter-inducing interferon-beta (TRIF), and TRAM. These adaptors activate a variety of signaling pathways. Refer to Figure 2 for a more detailed description of TLR-signaling pathways.

High mobility group box 1 (HMGB1) is a protein that in humans that is encoded by the HMGB1 gene. Platelets bind to HMGB1 but the cell surface receptor mediating this interaction is less documented. Platelets express previously recognized HMGB1 receptors TLR2/4/9, RAGE, transmembrane proteoglycans, and anionic lipids. Whether these structures mediate HMGB1 binding to platelets has not been much studied.

Recently, Yu et al. (100) evidenced a mechanism by which platelets promote tumor cell metastasis and suggest TLR4 – and its endogenous ligand HMGB1 (alarmin HMGB1) – as targets for antimetastatic therapies. The Manfredi’s team reported that activated platelets present HMGB1 to neutrophils and commit them to autophagy and neutrophil extracellular trap (NET) generation (101); further, the abundantly produced ROS dramatically increased the ability of extracellular HMGB1 to activate blood leukocytes (102). Moreover, Vogel et al. (103), demonstrated that migration of mesenchymal stem cells (MSC) to apoptotic cardiac myocytes and fibroblasts was driven by hepatocyte growth factor (HGF), and platelet activation was followed by HMGB1/TLR4-dependent downregulation of HGF receptor MET on MSC, thereby impairing HGF-driven MSC recruitment.

Toll-like receptors are vital to immunity. However, inappropriate responses can, alternatively, trigger chronic and acute inflammation as well as auto-immune illnesses (triggered by the recognition of endogenous ligands) (104).

### TLR expression on/in megakaryocytes and platelets

#### Identification in megakaryocytes

Megakaryocytes (MK) have been shown to contain mRNA, which codes for TLRs, consistent with these receptors being continuously expressed in MK lineage cells rather than captured through the circulation (34, 35, 62). Moreover, several studies have shown TLR expression both on human megakaryocyte lineage cells (34) and on the MK of mice or isolated from human donors (62, 105), suggesting the origin of platelet TLR expression.

Toll-like receptor 4 expression increases during the MK maturation process. The kinetics of expression of this receptor is similar to that of CD41 (106). Similarly, TLR9 shows a considerable increase in the number of transcripts from day five of MK differentiation in pro-platelets (105).

In contrast to the burgeoning studies into the role of platelet TLRs, few studies have been conducted on the functional role of TLRs on MKs. Two studies (44, 106) have shown that TLR4^−/−^ mice have defects in their circulating and reticulated platelet counts compared with wild-type mice, suggesting that TLR4 could play a role in thrombocytopoiesis. Recent studies have shown that hematopoiesis is not a stereotypical phenomenon; rather, it can be activated by an inflammatory environment (107). The TLR4 of hematopoietic precursors may be involved in this regulation. This hypothesis appears to be confirmed by a study showing that mice stimulated by a TLR4 ligand, at a non-lethal dose, have a higher number of platelets compared with untreated mice (44). An increase in the number of encoding transcripts has also been noted for TLR1 and TLR6 in MKs grown in the presence of IFNγ in both a dose-dependent and a time-dependent manner (107). This could be a mechanism that enables newly formed platelets to be more numerous, augments the amount of their TLRs and plays a more active anti-infectious role as a result.

#### Early identification on/in platelets

**Figure 3:**
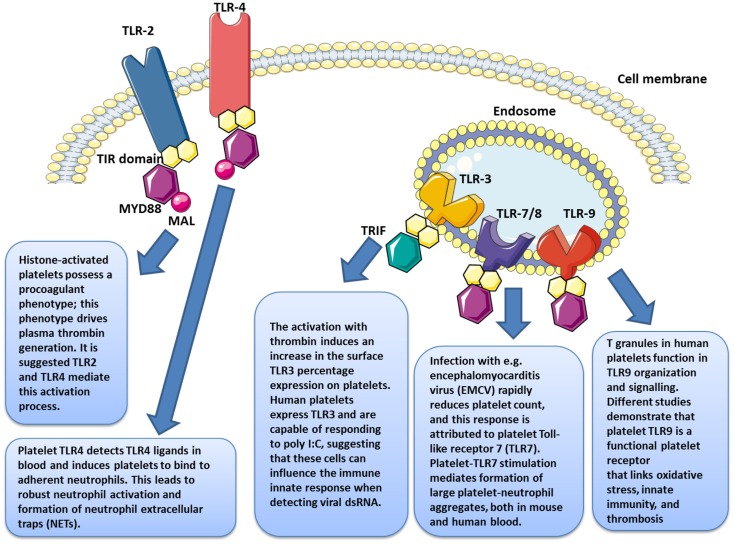
**Platelets express several functional toll-like receptors (TLRs), such as TLR2, TLR3, TLR4, TLR7, and TLR9, which may potentially link innate immunity**.

In 2004, Shiraki et al. demonstrated the presence of TLR1 and TLR6 on platelet surfaces and their possible involvement in the process of atherosclerosis (34). Studies conducted that same year by our team complemented these findings with the discovery of TLR2, TLR4, and TLR9 on both the human platelet surface membrane and in the cytoplasm; further these TLRs could be modulated, based on the state of platelet activation (106, 108, 109) (Figure 3).

Platelet TLR expression has also been observed in rodents (62), which are a widely used model for the study of these molecules; the characterization of TLRs has also been investigated on the surface of in chicken thrombocytes (110).

#### Function of TLR4 in platelets

Toll-like receptor 4 is the most abundantly expressed TLR on platelets (111). Several groups have examined its function in humans and mice (10, 19, 35, 106, 109).

Lipopolysaccharides (LPS), a major component of the membrane of Gram-negative bacteria, are ligands of TLR4. On eukaryotic cells, TLR4 forms a complex with the MD-2 molecule, thus enabling binding to LPS. Other proteins contribute to LPS binding, such as the “LPS Binding Protein” (LBP) and CD14 (98, 112).

The engagement of platelet TLR4 by LPS significantly increases the number of spliced mRNA encoding tissue factor (TF), which are then translated into functional proteins (113). LPS has been shown to cause severe thrombocytopenia in murine models, with a 60% decrease in the platelet count 4 h after treatment with LPS, compared with a 20% decrease in TLR4 KO mice (106). These results, therefore, suggest that platelet TLR4 is functional *in vivo*. The reduction in circulating platelets after LPS binding can have two causes: (i) an accumulation of platelets in the lungs (along with the otherwise described neutrophil sequestration) (52) and (ii) an increase in their destruction by phagocytosis (114, 115).

The signaling through TLR in general and TLR4 in particular, in platelets has also been examined. Specifically in platelets, the associated intracellular signaling pathways are less clear than in nucleated cells, as the purpose and function of these transcription factors has to be further elucidated. However, the engagement of platelet TLR4 potentiates signaling pathways traditionally detected in platelets. A notable example is the LPS dose-responsive increase in cGMP (116). Inhibition of PKG signaling blocks TLR4-dependent platelet responses, suggesting that cGMP is involved in TRL4-dependent activation (117). MyD88 expression has also been demonstrated in platelets at levels comparable to that obtained with leukocytes (117). Moreover, LPS-induced aggregation was not observed in MyD88-deficient mice or using salting-out processes in immunomodulatory factors (117). As is the case with other cell types, MyD88 is required for transmission of the signal emitted by TLR4 in platelets. It may be proposed, therefore, that either there is the creation of a link with other signaling pathways or the initiation of a TLR pathway specific to platelets. This suggestion is based on our observations that in addition to MyD88, platelets express most of the molecules traditionally involved in signal transduction, such as TRIF, MyD88, TBK-1, IRAK-1, JNKs, MAPk, TRAF3, TRAF6, IRF-3, IKK-i, IκB-α, and NF-kB p65 (111). For an identical amount of protein extracts, the expression of certain molecules is at times even stronger in platelets than in PBMCs. The level of signal transduction molecules in platelets is quite abundant, in amount that would be usable for intracellular signaling in platelets; the precise meaning of such abundant levels of, i.e., NF-kB in platelets is questioned. This would explain why we showed a difference in platelet activation depending on whether a smooth-type or a rough-type LPS is used as the stimulant. Studies in leukocytes revealed that smooth-type LPS stimulates only the MyD88 pathway while rough-type LPS, owing to its high affinity, has the ability to activate both pathways. While this distinguishing mechanism is present in platelets, the activation of each of these pathways proved to be associated with differential cytokine release (118). TRAF6 has a key role in signaling via platelet TLR4, leading possibly to de novo synthesis of IL1-β. In fact, direct stimulation of TRAF-6 produces splicing five times thicker than is obtained in platelets stimulated by LPS. TRAF-6 activation is followed by phosphorylation of Akt and JNK (119). These phosphorylation events are sufficient to initiate the splicing of mRNA encoding IL-1β. Consequently, a derivation of the TLR4 pathway by TRAF6 occurs, allowing the signal to pass through the Akt and MAP kinase pathways.

Several studies have reported that TLR4 expression varies depending on the state of activation of the platelet (23, 109, 120) (Figure 3). Platelet stimulation by thrombin leads to increased TLR4 expression, which, in synergistic combination with membrane CD62P, enables improved LPS binding (120). The fact that platelets have an intracellular pool of TLRs (108) may account for the receptor’s translocation to the surface during activation. Even while the LPS does not apparently induce platelet aggregation, it nonetheless potentiates the response triggered by very small concentrations of thrombin and collagen. Our group has shown that TLR4 engagement potentiates the activation of cGMP, bridging activation and aggregation; however, when used at high doses, LPS appears to induce aggregation by itself (57).

As in eukaryotic cells, the function of platelet TLR4 is supported by one or more plasma molecules; the addition of recombinant CD14 to washed platelets, as well as LBP, allows optimal activation to be restored (119–121). CD14, and possibly also MD-2, could be borrowed by platelets in the environment. This is, however, somehow disputed (57).

Several studies have examined the effect of platelet TLR4 engagement on the salting-out of immunomodulatory molecules. For example, Aslam et al. demonstrated that the administration of *Escherichia coli* O111 LPS to mice leads to an increase in the serum levels of “Tumor necrosis factor” (TNF)-α, mainly derived from platelets (109). Our team used similar LPS to stimulate *in vitro* purified platelets obtained from healthy blood donors; we observed a modulation of certain molecules: some were increased (sCD40L and PF4) while others remained unchanged (sCD62P, IL-8, EGF, and TGF) or were even reduced (RANTES, angiogenin, and PDGF-AB) (122). Platelets thus have a highly regulated system for the release of cytokines.

Furthermore, it has been shown that platelets can distinguish between two types of LPS, referred to as “smooth” and “rough,” and to adapt their cytokine response accordingly. In this way, only the smooth-type LPS molecules significantly inhibit the secretion of PDGF-AB (118). In contrast, rough-type LPS, unlike smooth-type LPS, potentiates the production of PMPs as well as platelet aggregation induced by the PAR1 agonists SFLLRN (123). The differences in activation can also be seen in the ability of the supernatants obtained to activate peripheral blood mononuclear cells (PBMCs) (118).

#### Function of TLR2 in platelets

The functional role of platelet TLR2 has not been as widely examined as TLR4. However, TLR2 is a highly inflammatory receptor that can recognize a very large number of PAMPs.

Among these ligands are, *inter alia*, bacterial lipopeptides, peptidoglycan, and lipoteichoic acid from Gram-positive bacteria, microbacterial lipoarabinomannan, yeast zymosan, and even viral hemagglutinin (89). TLR2 forms heterodimers with TLR1 or TLR6: the TLR2/TLR1 complex preferentially binds triacylated lipopeptides (Gram-negative bacteria and mycoplasma), and the TLR2-TLR6 complex binds diacylated lipopeptides of Gram-positive bacteria and mycoplasma.

Structural studies are consistent with the mechanism for such discrimination involving the presence of a hydrophobic channel present on the TLR1 but not on the TLR6 (89).

The engagement of platelet TLR2 has also been reported to stimulate the splicing of mRNA encoding IL-1β (121). However, this synthetic lipopeptide is less effective than LPS, though just as effective as thrombin. The splicing of mRNA encoding IL-1β can, therefore, be regulated differently depending on the TLR activated.

Regarding the signaling pathway, TLR2 uses MyD88 to commence its signaling cascade (124). Platelet TLR2 also has the ability to trigger the activation of signaling proteins normally involved downstream of hemostatic receptors. Morello et al. demonstrated that platelet TLR2s were functional and showed that the PI3K/Akt pathway (already detected in platelets, downstream of αIIbβ3) plays a role in platelet aggregation, adhesion, and secretion (125). They further demonstrated that in immune cells, TLR2 has the ability to interact with the p85 sub-unit of PI3K (126), suggesting the involvement of this pathway after TLR2 engagement, independently of the MyD88 pathway. The use of a specific inhibitor of PI3K (LY294002) prior to stimulation by Pam3CSK4 significantly reduces the effects generated by the engagement of platelet TLR2 (i.e., the aggregation, adhesion and membrane expression of CD62P, platelet-neutrophil complex formation, αIIbβ3 activation, as well as oxygen radicals) (127). LY294002 also significantly diminishes, but does not completely inhibit, the formation of platelet:neutrophil aggregates, suggesting that platelets can use alternate pathways besides TLR2 and 4. Other studies showed that during platelet stimulation by *Aggregatibacter actinomycetem comitans Y4* and *Porphyromonas gingivalis*, the salting-out of sCD40L resulting from TLR2 engagement is regulated by PI3K (128). However, PI3K is not the only pathway involved in the release of soluble factors induced by TLR2 engagement. In fact, the use of a PLC inhibitor, U73122, prevents CD40L from being expressed on the platelet surface in a manner similar to that observed when the PI3K pathway is blocked (128). PLC is classically identified in the platelets as acting downstream of PAR and GP-VI receptors, leading to degranulation and the generation of TxA_2_.

Phospholipase C and P3IK pathways are, therefore, not only activated by hemostatic stimulation but also following TLR2 engagement. However, phosphorylation kinetics differs depending on the type of stimulation involved (129). In this way, stimulation by thrombin induces a rapid and substantial phosphorylation of Akt, p38, and Erk. In contrast, stimulation by Pam_3_CSK_4_ induces a more gradual phosphorylation of Akt and p38, suggesting that it takes longer for the mechanisms to occur and that platelet inflammatory responses take place over time rather than rapidly. The large majority of experimental studies to stimulate TLR2 on platelets use Pam_3_CSK_4_, a triacylated ligand that causes the dimerization of TLR2 with TLR1. The use of MALP-2, a diacylated ligand that makes use of the TLR2/6 heterodimer, does not allow platelet activation (124). In addition, the preincubation of platelets with MALP-2 reduces and even inhibits the effect of Pam_3_CSK_4_. The engagement of TLR2 and TLR6 is believed rather to have an antagonist effect on platelet activation and could act as a regulator in platelet activation during bacterial invasion by simply blocking access to TLR2.

The first demonstration of platelet TLR2 function, *in vivo* as well as *ex vivo*, was reported in 2009 by Blair et al. (127). The stimulation of platelets by Pam_3_CSK_4_, a synthetic ligand mimicking bacterial lipopeptide, caused activation involving an “inside-out” αIIbβ3 signaling, aggregation, and platelet adhesion to collagen, CD62P release and generation of reactive oxygen derivatives. These phenomena were either blocked by a TLR2 antagonist antibody or absent in *tlr2*^−/−^ KO mice, demonstrating the engagement of the receptor. Another study on the functional role of platelet TLR2 showed that Pam_3_CSK_4_ also controls increases in platelet intracellular Ca^2+^ concentrations, the release of ATP, and the synthesis of TxA_2_ (124). These observations are consistent with a role for platelet TLR2 as a thrombo-inflammatory receptor. Even where the ultimate thrombotic purpose is similar to that of classic stimulation (ADP, Fg, collagen, etc.), the intracellular mechanisms activated are quite distinct, particularly with regard to the phosphorylation of signaling molecules (PI3K and MAPK pathways) and protein–protein interactions (mainly in the case of the FXIIIa protein involved in platelet remodeling). Compared with hemostatic stimulation, there are also differences in the release of granular proteins (Fg, thrombospondin, PF4) which can be released or sequestered during TLR2 stimulation (130).

Apart from the thrombotic function of the platelet TLR2, this receptor can also induce an inflammatory response by platelets. Bacterial stimulation of platelets by periodontopathogens (*A. actinomycetemcomitans* and *P. gingivalis*) demonstrated that sCD40L is released independently of TLR2. Furthermore, patients who have undergone restorative dental procedures had a significantly higher level of circulating sCD40L than healthy control subjects (128).

It has been observed, moreover, that mice infected with *P. gingivalis* displayed a higher proportion of platelet-neutrophil complexes than uninfected mice or TLR2^(−/−)^ mice, suggesting that platelet TLR2 is involved in the formation of platelet-neutrophil aggregates (127).

Assinger et al. confirmed these observations and showed that this increase in platelet–neutrophil complexes is accompanied by a rise in neutrophil-mediated phagocytosis of periodontopathogens, which requires TLR2 to be functional (131). Platelet TLR2 is thought to be a prerequisite to the activation of the latter, which are then believed to transmit a signal activating the neutrophils and making them suitable for phagocytosis. The particular signal can involve ligand:receptor pairs (CD62P/PSGL-1, CD40L/CD40, GP-IIb–IIIa/CD11b) or platelet cytokines.

Similarly, it has recently been observed that platelets promote the clearance of *Staphylococcus aureus* and *Bacillus cereus* by Kupffer cells.

When absorbed by these liver macrophages, such bacteria are thought to be rapidly engulfed by a platelet aggregate, predisposing them to destruction. The formation of aggregates would involve the participation of platelet GP-Ib and vWF on Kupffer cells (32).

The release of histones from cells in apoptosis is associated with microvascular thrombosis. In citrated PRP, the presence of histones has been proven to stimulate thrombin generation, independent of dose, with noticeable effects from 10 μg/ml (132). When an anti-TLR2 monoclonal antibody is used prior to stimulation by histones, the procoagulant profile of platelets [membrane expression of phosphatidylserine (PS), CD62P, and coagulation factor V] is reduced. Under the same conditions, TLR2 blockade leads to a reduction of about 50% in the salting-out of thrombin and increases the salting-out time for the remaining 50% by 40 minutes (Figure 3).

Platelet TLR2 is, therefore, involved in histone-induced thrombin generation. These results reaffirm the importance of platelet TLR2 in a thrombo-inflammatory response. Inversely, this response can be considered beneficial to the host in cases where the agent that induces cell apoptosis becomes trapped in the fibrin network.

#### Function of TLR9 in platelets

Studies conducted on the role of platelet TLRs rarely address the subject of TLR9. In eukaryotic cells, TLR9 recognizes unmethylated 2′-deoxyribo (cytidine-phosphate guanosine) DNA motifs, commonly referred to as CpG motifs, which are found specifically in bacteria, parasites, and viruses. The location of TLR9 is limited to the endosomes in eukaryotic cells, enabling it to recognize the internal constituents of pathogens, which are often released after they are endocytosed (89, 133).

Platelet TLR9 is also found in cell cytosol and cell membranes (108). Activated platelets overexpress this receptor, either after stimulation by a CpG motif (105) or by thrombin (109).

A recent study on human and murine platelets showed that intra-platelet TLR9 is distributed in a specific, previously unidentified, sub-compartment known as the T granule, which has dense appearance under an electron micrograph (105, 134). This distribution of TLR9 is thought to occur during pro-platelet formation. T granules also contain VAMP7 and VAMP8 proteins, which are involved in directing TLR9 to the membrane. The process by which platelets internalize the CpG/TLR9 complex appears to be similar to that described for other cell types (105, 134).

Platelet TLR9 was recently reported as binding carboxy-alkyl-pyrrole, a product derived from the combination of polyunsaturated fatty acid oxidation products and protein products, considered a danger signal in cases of oxidative stress (135). In platelets, this joining together triggers aggregation and degranulation. Therefore, in platelets TLR9 appears to function as sensors of internal danger signals rather than external ones (Figure 3).

It is only recently that TLR3 (37) and TLR7 (38) have also been identified (Figure 3). Human platelets express TLR3 and are capable of responding to poly I:C, suggesting that these cells might influence the immune innate response when detecting viral dsRNA (37). Infection with encephalomyocarditis virus (EMCV) rapidly reduces platelet count, and this response is attributed to platelet Toll-like receptor 7 (TLR7) (38).

## The Siglec: Sensors of Pathogens Newly Acknowledged on Platelets

### Background on Siglec molecules

Siglec molecules belong to the large family of Ig-like lectins (84, 87, 96), which are categorized in two main families (Table 2). They are type I transmembrane proteins comprising three regions (84, 86, 136). First, the extracellular region has a “V-set” N-terminal Ig domain containing an arginine residue that forms a saline bridge with the carboxylate group of sialic acid, which enables specific binding with this molecule and a number varying between 1 and 16 in the “C2-set” domain (97, 136). Second, the transmembrane region allows the signal to be transmitted to the third region, the intracellular region of the receptor, which contains an immunoreceptor tyrosine-based inhibitory motif (ITIM) close to the membrane, and an ITIM-like motif, which is more distal from the membrane. Certain Siglec molecules, such as sialoadhesins (137), Siglec-H (138), Siglec-14 (139), Siglec-15 (140), and Siglec-16, do not have an intracellular region (141).

The primary function of CD33r Siglec (3, 5–11, 14, 16) is to recognize “self” molecules in order to regulate host immune response by the engagement of inhibitory ITIM-like intra-cytoplasmic molecules. Siglec principally recognize and bind sialylated glycans and gangliosides.

Siglec molecules have arginine residues that can form a saline bridge with the carboxylate group of sialic acid enabling them to bind in a specific way (136). In their basal state, Siglec are bound with ligands and are, therefore, expressed on the same cell (*cis* interaction); thus their binding sites are generally masked (84, 95). The interaction is, therefore, predominantly a *cis* interaction (95). However, *trans* interaction may compete with *cis* interaction when the sialylated (*trans*) glycan ligands are attached to glycoproteins and glycolipids, which gives them higher affinity [for example, PAMP structures (LPS or bacterial peptidoglycan) or gangliosides] (96). Furthermore, the *cis* interaction sites can be cleaved by sialidase [a component of certain pathogens like *Vibrio cholerae, Clostridium perfringen*, and *Arthobacter ureafacien* (96, 97)], or unmasked following cell activation, which enables Siglec to have *trans* interactions (142).

The gangliosides formed from glycosphingolipids are bound to Siglec with a very high affinity (143). The affinity of CD33r Siglec is different for each ganglioside depending on their structure; for example, Siglec-7 and Siglec-9 are selective in targeting B-series gangliosides containing the 2,8-disialyl residue (GalNAc or GlcNAc), such as GQ1b, GT1b, GD2, and GD3, while Siglec-8 appears to have low affinity with these gangliosides (143–146).

The main known function of Siglec molecules are:

(i) the regulation of innate immune responses by balancing (down: *cis* ligation; up: *trans*-ligation) the defense against a number of infectious pathogens that use sialic acid to disguise themselves in an attempt to escape elimination by the infected body;
(ii) the negative regulation of the B-cell receptors (by co-engagement of two main Siglec: CD22 and Siglec-10 [Human; -G in the mouse; no cluster of differentiation assigned so far] (84, 87, 95, 96, 143, 147) and of T-cell receptors [by co-engagement of three main Siglec: 7, 9 and 1 [sialoadhesin] (136)].

Siglec molecules operate by phosphorylating principally ITIM/ITIM-like motifs in their intracellular tails, or – exceptionally (Siglec-H and -15) – by mobilizing DAP12. Thus, Siglec appear in general essential to preventing excessive innate and adaptive immune responses and maintaining homeostasy and tolerance.

### Siglec and platelets

A substantial expression of Siglec-7 has been identified very recently (by our group) on platelet surfaces, with more stored on platelet α-granule membranes. Furthermore, surface membrane expression of Siglec-7 is significantly increased after platelet activation, in a manner similar to the activation-induced membrane expression of CD62P. Indeed the kinetics of Siglec-7 expression on the platelet membrane of closely resembles that of CD62P.

There is also a significant amount of Siglec-7 in the supernatant in both control and TRAP-activated platelets, consistent with the cleavage, or direct salting-out, of Siglec-7 from platelet α-granules. However, Siglec-7 cleavage does not correlate with a reduction in its expression on the platelet membrane, as is the case for CD62P. A probable explanation for this apparent reduction in concentration over time may be the degradation induced by endogenous proteases.

The engagement of Siglec-7 by its specific ligand, GD2 ganglioside (as well as GD3 and GT1b), does not induce activation, aggregation, or platelet secretion, but leads to platelet apoptosis by the intrinsic and an extra-mitochondrial pathway. Similarly, Martini et al. demonstrated that platelet incubation with exogenous GD3 had no effect on platelet morphology, nor function, nor on ADP-induced platelet aggregation (148). The authors also indicated that GD3 functions like a second-messenger molecule that augments CD32 expression (FcgRII, platelet FcR isoform); it then binds to this receptor, leading to platelet adhesion on the subendothelial matrix (148). In our study, however, the apoptogenic effects of GD2 on platelets were independent on the engagement of CD32, whereas reduced in the presence of specific antibodies that block Siglec-7. This shows that Siglec-7 engagement is essential for GD2-induced platelet apoptosis. Inhibitors of NADPH oxidase, PI3k and PKC, but not of NF-kB prevented. The engagement of the P2Y1 platelet receptor and of the GP-IIb–IIIa integrin is required for a fully functional Siglec-7.

Platelet apoptosis induced by Siglec-7 engagement is probably a mechanism for negatively regulating platelet inflammatory responses. This mechanism would limit excessive reactions responsible for the destruction of tissues and cells due to inflammation and in this way promotes the healing of wounds. Stored platelets (with the purpose of being used for transfusion purposes) display apoptosis markers that increase independently on activation (149–151). This is a significant aspect of storage damage that reduces the viability and number of platelets in PCs following prolonged storage. Platelet apoptosis can potentially have negative effects in PC recipients, such as reduced function and altered corrected count increment (CCI) (149); probable adverse effects due to the pro-inflammatory and pro-thrombotic properties of PMPs generated during apoptosis are also likely (152).

The mechanisms of Siglec-7 translocation to human platelet membranes and its subsequent cleavage are still unaddressed (88) and require further studies. It would be interesting, first, to discover whether this increase is an advance event for triggering platelet activation or whether it is secondary, occurring after platelet activation in order to negatively control inflammatory response following this process (autoregulation). Second, it will be important to determine whether cleavage of soluble Siglec-7 is associated with such translocation. Little is known of the role of soluble Siglec-7 in inflammatory reactions, particularly in immune cells, ECs and platelets, or of the importance of the physiology and platelet interactions at work in the immune system.

In addition, it would be interesting to be able to determine whether Siglec-7 engagement is also specifically linked to platelet recognition of DAMPs. In other words, it would be valuable to examine whether platelet Siglec-7 distinguish several PAMPs (infectious pathogens). Moreover, it would be interesting to investigate platelet Siglec-7 response after DAMP activation (generated either during PAMP-induced inflammatory reactions or due to intra-platelet oxidative stress linked to preparation and/or storage conditions) and how their inflammatory response is modulated. Several studies show that by expressing a very large number of receptors, among them TLRs, GPCRs, RTKs, integrins, and CK/CH receptors, platelets appear to have the ability to differentiate between a hemostatic stimulus or an infectious stimulus, and endogenous danger signals (DAMPs) and exogenous ones (PAMPs). This method of differentially detecting pathogens produces variations in membrane expression of adhesion molecules, activation molecules, and especially in the salting-out of platelet granule contents and their secretion kinetics, leading to various platelet responses, such as hemostatic, inflammatory, or reparative (35, 118, 122, 153, 154). Furthermore, it would be interesting to study Siglec-7 in the platelet signalosome in the presence of various stimuli, including hemostatic stimuli (as shown in the appendix), infectious agents (PAMPs), or DAMPs. To this effect, numerous studies are considered to complement the characterization of the functional role of platelet Siglec, including Siglec-7.

**Figure 4:**
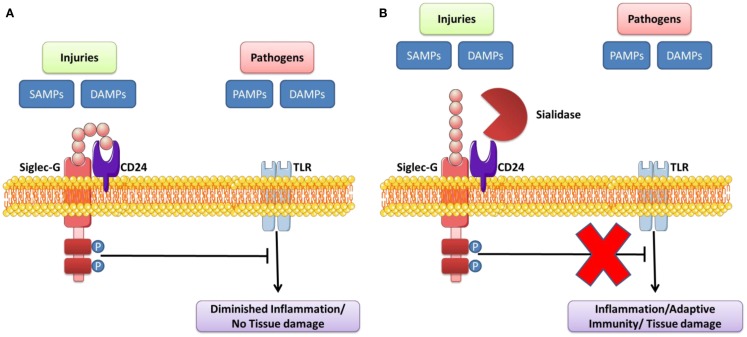
**Sialidase interrupts the Siglec-G inhibitory function that suppresses TLR signaling by DAMPs/PAMPs**. **(A)** CD24 forms trimolecular complex with DAMPs/SAMPs and Siglec G that inhibits activation of TLR. **(B)** Pathogen-encoded sialidases cleave sialic acids on CD24 from interacting with Siglec G, leading to induce the inflammation/adaptive immunity/tissue damage. Adapted from Ref. (95, 142, 157).

Siglec molecules contribute to the negative regulation of the intracellular pathways stimulated following TLR/NLR engagement. This serves to prevent excessive immune responses after activation of these receptors (96). The regulation and activation of cells after TLR engagement is different for each cell type (95). This inhibitory function of Siglec is used by pathogens to imitate the structure of their ligands, which initiates an immune response favorable to their survival within the host. Recent studies show that, when Siglec molecules are neutralized by blocking antibodies, TLR signaling is significantly modified; this suggests that the density of Siglec expression near to TLRs can influence the function of these receptors (95). The location (on membranes or endosomes) where Siglec sensors are expressed can also modulate innate immune response in the host following TLR engagement (Table 3). Siglec molecules are normally expressed on immune cells that contribute to regulation of the innate immune system (96). PRRs can recognize “self” (DAMP) and “non-self” (PAMP) danger signals in order to trigger inflammatory reactions. The engagement of these receptors after engagement by DAMPs can also reduce inflammation and promote the repair and healing of wounds (142). Chen et al. originally illustrated the mechanism by which Siglec-10/Siglec-G enables innate immunity cells to distinguish DAMPs and PAMPs in order to set up an immune response that can defend against pathogens, while at the same time preserving the integrity of tissue at the end of the post-infection defense process. They showed that the interaction between CD24 and human Siglec-10 (murine Siglec-G) can reduce inflammatory response induced by DAMPs of the HMGB1 and HSP70 types, but not in the presence of certain PAMPs (LPS and PolyI:C which activate TLR4 and TLR3, respectively). The glycoprotein CD24 actually has the ability to form a complex of Siglec-10/Siglec-G and DAMP, which inhibits TLR/NLR activation by inhibiting ITIM motifs (Figure 4). However, certain infectious pathogens like *Vibrio cholerae, Clostridium perfringen*, and *Arthobacter ureafacien* (96, 142), which have a sialidase, can cleave the bond between Siglec sensors and CD24; in this case, the distinction between the DAMP and PAMP signals will, as a result, not be effective.

**Table 3:** **Modulation of TLR function by Siglecs adapted from Ref. (95, 155, 156)**.

Molecules	TLR ligands used	Cell type	Observed phenotype
CD22	TLR3, 4, 7, and 9	B	Enhanced proliferation of CD22 KO B cells
Siglec-G	TLR3, 4, 7, and 9	B	Enhanced proliferation of Siglec-G KO B cells
	HMGB1	DC	Enhanced TNF-α production in Siglec-G KO DCs
Siglec-E	TLR4	Mac	Reduced IL-12 production by cross-linking with Abs
Siglec-H	TLR9	pDC	Reduced IFN-α production by cross-linking with Abs
Siglec-5	TLR2, 3, 4, and 9	Mac	Reduced TNF-α and enhanced IL-10 production by over-expression
Siglec-9	TLR2, 3, 4, and 9	Mac	Reduced TNF-α and enhanced IL-10 production by over-expression
Siglec-11	TLR4	Mac	Reduced IL-1β transcript by cross-linking with Abs
Siglec-14	TLR4	Mac	Augmented TNF-α production by over-expression
CD33/Siglec-3	TLR4/CD14	imDCs	Reduced the phosphorylation of NF-κB
Siglec-9	TLR2	Mac	Siglecs exhibit lectin-dependent changes in cellular localization, which may be partly linked to its control mechanism that increases the production of IL-10

## Conclusion

As a conclusion, analyzing platelet responses to PRR agonist stimulation in whole blood, platelet-rich plasma and in transfusion platelets concentrate would help clarify the relative contributions of platelets on inflammatory process. How can the small platelet, without a nucleus, be so intelligent?

## Conflict of Interest Statement

The authors declare that the research was conducted in the absence of any commercial or financial relationships that could be construed as a potential conflict of interest.
